# Differentiation of Insulin-Producing Cells from Human Neural Progenitor Cells

**DOI:** 10.1371/journal.pmed.0020103

**Published:** 2005-04-26

**Authors:** Yuichi Hori, Xueying Gu, Xiaodong Xie, Seung K Kim

**Affiliations:** 1Department of Developmental Biology, Stanford University School of MedicineStanford, CaliforniaUnited States of America; **2**Department of Medicine, Oncology DivisionStanford University School of Medicine, Stanford, CaliforniaUnited States of America; Albany Medical CollegeUnited States of America

## Abstract

**Background:**

Success in islet-transplantation-based therapies for type 1 diabetes, coupled with a worldwide shortage of transplant-ready islets, has motivated efforts to develop renewable sources of islet-replacement tissue. Islets and neurons share features, including common developmental programs, and in some species brain neurons are the principal source of systemic insulin.

**Methods and Findings:**

Here we show that brain-derived human neural progenitor cells, exposed to a series of signals that regulate in vivo pancreatic islet development, form clusters of glucose-responsive insulin-producing cells (IPCs). During in vitro differentiation of neural progenitor cells with this novel method, genes encoding essential known in vivo regulators of pancreatic islet development were expressed. Following transplantation into immunocompromised mice, IPCs released insulin C-peptide upon glucose challenge, remained differentiated, and did not form detectable tumors.

**Conclusion:**

Production of IPCs solely through extracellular factor modulation in the absence of genetic manipulations may promote strategies to derive transplantable islet-replacement tissues from human neural progenitor cells and other types of multipotent human stem cells.

## Abstract 2

The search for cells that can secrete insulin need not be limited to pancreatic precursors; cells derived from neural progenitors can also produce insulin and respond to glucose

## Introduction

Advances in transplant-based therapy for type 1 diabetes mellitus [1] and a dearth of cadaveric pancreatic islets of Langerhans have focused interest on developing renewable sources of transplant-ready islet-replacement tissues. Pancreatic islets derive from embryonic endoderm, but display features of neurons, including a shared set of cell-autonomous developmental regulators [2]. Islets are the principal source of insulin in humans, but in some invertebrate species, such as *Drosophila,* brain neurons are the main source of circulating insulin [3,4]. The similarities between islet cells and neurons are further underscored by the demonstration of insulin gene transcription in the vertebrate brain [5], although it remains unclear whether these vertebrate neurons produce or secrete insulin protein [6]. Moreover, recent studies also suggest that in some settings, mouse pancreatic epithelial cells can engender neuron-like cells [7,8]. These and other findings [2] suggest that common, ancient developmental programs may govern the differentiation of islet cells and neurons. Methods promoting neural differentiation by embryonic stem (ES) cells have been adapted [9,10,11,12,13] to derive insulin-producing cells (IPCs), but it remains unclear whether such IPCs were derived from neural progenitors, or whether a neural-based strategy can generate transplantable human IPCs.

Neural cells derive from multipotent progenitor cells, and recently, clonal human neural progenitor cells that remained multipotent and karyotypically stable through at least 15 passages were purified by flow-cytometry-based methods [14]. The availability of human neural progenitor cells allowed us to investigate whether inductive signals involved in normal pathways of islet development could direct these neural progenitors to develop into glucose-responsive IPCs. Here we describe experimental strategies for developing IPCs directly from human neural stem cells.

## Methods

All studies were performed in accordance with appropriate human participants' approval according to Stanford University Institutional Review Board guidelines.

### Cell Lines and Culture Conditions

The human neurosphere (NS) cell lines (line numbers 1651, 1664, and 1673) were established following enrichment for AC133^+^ cells, as previously described [14], although none of the cell lines was expanded from a single cell, and they are therefore not clonal. The different cell lines were from distinct donors and karyotypically normal. They gave comparable results, but line 1664 produced less insulin by stage 4 in our protocol than the other cell lines. Following cell thaw, NSs were expanded in suspension culture, and in each case were used between passages 5 and 7. Undifferentiated human NS cells (stage 1) were cultured and expanded in medium containing X-VIVO15 (Cambrex, Walkersville, Maryland, United States), N2 supplement (Invitrogen, Carlsbad, California, United States), 2 μg/ml heparin, 10 ng/ml leukemia inhibitory factor (Chemicon, Temecula, California, United States), 20 ng/ml epidermal growth factor (R&D Systems, Minneapolis, Minnesota, United States), and 20 ng/ml fibroblast growth factor-2 (R&D Systems), and they were frozen after enzymatic dissociation. At stage 2, we thawed and cultured 10^7^ cells in medium made from a 1:1 mixture of glucose-free DMEM (Invitrogen) and F-12 medium (Invitrogen) containing 0.2% bovine serum albumin (Sigma, St. Louis, Missouri, United States), N2 supplement, 2 μg/ml heparin, 10 ng/ml leukemia inhibitory factor, 20 ng/ml epidermal growth factor, and 20 ng/ml fibroblast growth factor-2. The final concentration of glucose in stage 2 was 5 mM. Cultures were fed weekly for up to 2 wk. Cells formed NSs during stages 1 and 2. After 2 wk, we transferred 40–80 NSs containing a total of approximately 2–4 × 10^5^ cells to single wells in plates coated with 0.005% poly-L-ornithine (Sigma) and 3 μg/ml fibronectin (Invitrogen). These were cultured for 2 wk (stage 3) in a 1:1 mixture of low-glucose DMEM and F-12 media containing 100 mg/l apo-transferrin (Sigma), 288 mg/l glucose, 73 mg/l L-glutamine, 1.69 g/l sodium bicarbonate, 25 μg/ml bovine insulin (Sigma), 20 nM progesterone, 100 μM putrescine, 30 nM sodium selenite, and penicillin/streptomycin. Stage 3 medium was supplemented with 2 μM all-*trans* retinoic acid (RA) (Sigma) and had a final glucose concentration of either 5 or 17 mM. At stage 4, cells were cultured in N2 medium [10] supplemented with 10 mM nicotinamide (Sigma) and 10 nM insulin-like growth factor-1 (R&D Systems) for up to 6 d. Stage 4 medium included 17.3 mM glucose. During stage 3 and 4, medium was changed every other day. To examine the effect of Sonic hedgehog (Shh) (R&D Systems), we added 300 nM mouse Shh in PBS every other day during stage 4. To quantify neurite outgrowths per cluster, 20 Shh-treated or control stage 4 IPC clusters were photographed under light microscopy, and digitized images were used to score neurites. Data are presented as the average ± the standard error of the mean. Two-tailed *t* tests were conducted to determine statistical significance.

### RT-PCR

Total RNA was prepared by using TRIZOL (Invitrogen) and RQ1 RNase-free DNase (Promega, Madison, Wisconsin, United States). For cDNA synthesis, random sequence primers were used to prime reverse transcription reactions and synthesis was carried out by Thermoscript RT (Invitrogen). A total of 35 cycles of PCR were performed using Platinum *Taq* High Fidelity DNA Polymerase (Invitrogen). GAPDH expression was used to normalize input template cDNA to analyze relative gene expression. Primer sequences and gene accession numbers are listed in Table 1. To confirm their identity, DNA products from PCR reactions were excised, cloned, and sequenced.

**Table 1:**
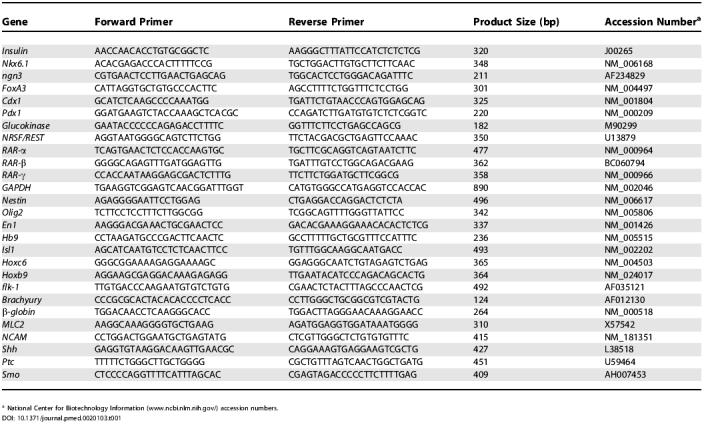
Specific PCR Primers and Gene Accession Numbers

^a^ National Center for Biotechnology Information (www.ncbi.nlm.nih.gov/) accession numbers

### Immunohistochemistry and Morphometry

Cell clusters were fixed in 4% paraformaldehyde, embedded in HistoGel (Richard-Allan Scientific, Kalamazoo, Michigan, United States), and then embedded in paraffin. We performed immunohistochemistry on 6-μm tissue sections. We used primary antibodies at the following dilutions: guinea pig anti-insulin, 1:200 (Linco Research, St. Charles, Missouri, United States), mouse anti-β-tubulin III, 1:500 (Sigma); rabbit anti-C-peptide, 1:100 (Linco Research); mouse anti-Nestin, 1:200 (Chemicon); mouse anti-Ki67, 1:100 (Novocastra, Newcastle, United Kingdom); rabbit anti–cleaved caspase-3, 1:200 (Cell Signaling, Beverly, Massachusetts, United States); rabbit anti-glucagon, 1:200 (Dako, Carpinteria, California, United States); rabbit anti-Glut-2, 1:200 (ADI, San Antonio, Texas, United States); mouse anti-proinsulin, 1:500 (O. Madsen, Hagedorn, Gentofte, Denmark); mouse anti-glucokinase, 1:200 (C. Newgard, Duke University, Durham, North Carolina, United States); mouse anti-GFAP, 1:1,000 (Dako); mouse anti-MAP2, 1:500 (Sigma); and rabbit anti-Olig2, 1:1,000 (H. Takebayashi, National Institute for Physiological Sciences, Okazaki, Japan). Confocal immunofluorescence microscopy with an optical slice thickness of 0.6 μm was performed on a Bio-Rad (Hercules, California, United States) MRC1000.

Cell counting and point-counting morphometry were performed using standard morphometric techniques [15]. To obtain representative results, all quantification of immunostaining was performed by counting numbers of positive-stained cells and dividing by the area of total tissues using a standard 10 × 10 microscope grid. For quantification of cells expressing Nestin, Ki67, β-tubulin III, activated caspase-3, insulin, or C-peptide, NS-derived cell clusters were fixed, and sectioned to generate 7-μm-thick tissue sections. Appropriately stained cells were counted in a minimum of ten random microscopic fields obtained from at least ten cell clusters per condition.

### In Situ Hybridization

We used human insulin cDNA (320 bp) cloned in plasmid pCR4-TOPO (Invitrogen) as a template for in vitro transcription to produce riboprobes with a digoxigenin-RNA labeling kit (Ambion, Austin, Texas, United States), performed hybridization with IPC cluster sections as described [16], and used DIG Nucleic Acid Detection Kit (Roche, Indianapolis, Indiana, United States) for development according to the manufacturer's instructions.

### Insulin C-Peptide Quantification and In Vitro Insulin Secretion Assay

At each stage, 300 NS or IPC clusters were handpicked, washed with PBS, and homogenized; then intracellular C-peptide content was measured with a C-peptide ELISA kit (American Laboratory Products Company, Windham, New Hampshire, United States). Stage 4 IPC clusters were cultured in glucose-free RPMI (Invitrogen) supplemented with 2.8 mM glucose, 20 mM HEPES, and 10% newborn calf serum (Invitrogen) for at least 2–3 h. At the end of these washes, no insulin was detectable in the wash supernatant using an ELISA kit (American Laboratory Products Company). Fifty IPC clusters were handpicked, transferred to a 24-well plate, and incubated for 2 h at 37 °C in the presence of 2.8 mM glucose, 25 mM glucose, or 25 mM sucrose. Supernatants were then harvested for enzyme-linked immunosorption assay (ELISA)–based quantification of released insulin.

### IPC Transplantation and Physiologic Tests

All animal studies were performed in accordance with Stanford University Animal Care and Use Guidelines. Under general anesthesia, 9- to 10-wk-old male NOD *scid* mice (purchased from Jackson Laboratories, Bar Harbor, Maine, United States) were engrafted with 1,000 handpicked IPC clusters in the right and left subcapsular renal space (500 IPC clusters each) or received a sham transplant of saline solution. Transplantation of more IPC clusters in a renal graft site was not feasible because of the size of the clusters. Two weeks after IPC cluster transplantation, we subjected mice to an overnight fast. Then we measured serum human C-peptide before and 30 min after intraperitoneal injection with 3 g of glucose per kilogram of body weight using a human C-peptide ELISA kit. When stage 4 IPC clusters were transplanted, tumors were not observed 4 wk after engraftment, the maximum period of observation (*n* = 5). All data represent the average (from the indicated number of samples) ± standard error of the mean. Two-tailed *t* tests were conducted to determine statistical significance.

## Results

### Glucose Restriction Initiates IPC Development

**Figure 1:**
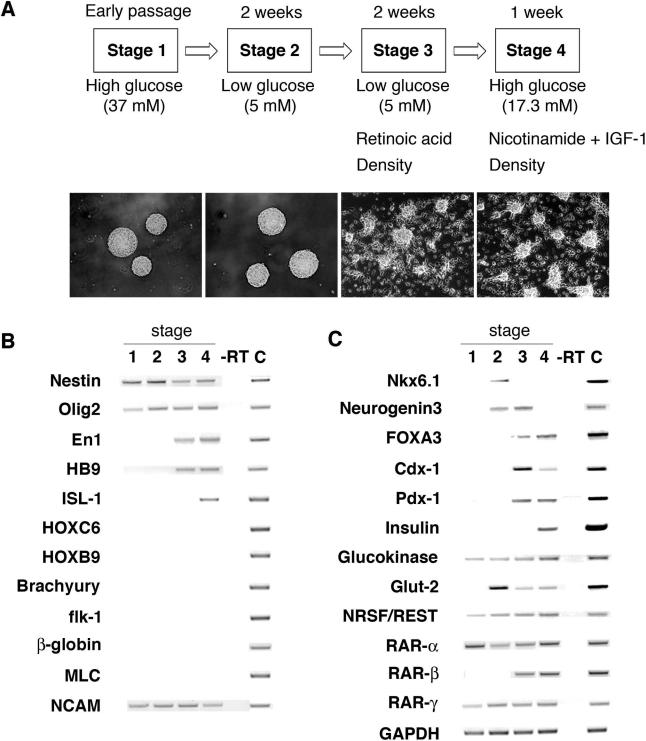
Development of Cells Expressing Islet Markers from Human Neural Progenitor Cells (A) Outline of four-stage differentiation protocol, essential factor manipulations at each stage, and stage-specific cell cluster morphology. Original magnification, 100×. (B) RT-PCR analysis of gene expression by stages 1–4 cell clusters, in stage 4 samples with RT omitted (–RT) and in human fetal spinal cord as a control (“C”). (C) RT-PCR analysis of expression during stages 1–4 of pancreatic and endodermal markers. Fetal pancreas RNA served as a control (“C”).

**Figure 2:**
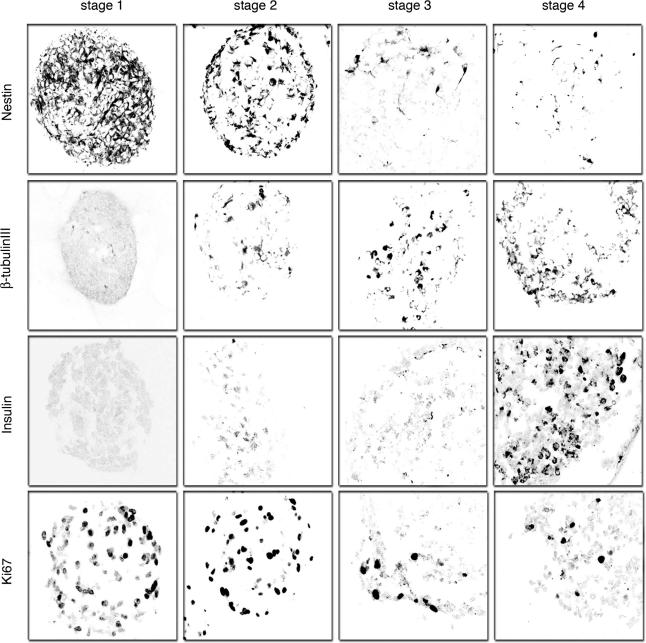
Immunohistochemical Detection of Neural Markers and Insulin during Stages 1–4 of Human Neural Progenitor Cell Differentiation Immunofluorescent images were obtained by confocal microscopy and are representative of at least ten samples for each antibody. We detected insulin in only stage 4 IPCs, consistent with RT-PCR results. Distribution of insulin staining is localized in the cytoplasm. Immunofluorescent detection of Ki67, a nuclear marker of proliferating cells, showed stage 4 IPCs are predominantly non-proliferating, similar to mature pancreatic islets. Original magnification, 630×.

Culture media with high glucose concentrations have been useful for isolating and maintaining progenitor cell populations from the central and peripheral nervous systems [14,17] and for permitting differentiation by neurogenic cells [18]. We routinely expanded NS cultures in medium containing 37 mM glucose, a level 5- to 6-fold higher than physiologic glucose concentration. Isolated human neural progenitors spontaneously aggregate in these conditions to produce a cell cluster called a NS, and 85%–90% of NS cells produce the intermediary filament Nestin (Figures 1 and 2), a putative marker of neural stem and progenitor cells [19]. Prolonged exposure to high glucose levels can also severely reduce insulin expression in pancreatic β-cells [20]. Thus, we postulated that glucose reduction might blunt neurogenic programs by NSs and promote alternate fates, including development of IPCs. Therefore, we switched NS culture conditions from high glucose (37 mM, “stage 1”) to low glucose (5 mM, “stage 2”; see Figure 1A) to initiate development.

To assess developmental changes resulting from glucose reduction and later culture modifications, we examined NS cell composition, and expression of gene products known to regulate or define in vivo fates of embryonic neural or islet cells. A β-tubulin III isoform produced in differentiating neurons was not detected in stage 1 cells, whereas expression of Ki67, an S-phase-associated nuclear antigen, was expressed in the majority of stage 1 cells (see Figure 2). Genes specifying transcription factors essential for in vivo differentiation of lineage-restricted neural progenitors and their differentiated progeny [21,22,23], including *En1, Hb9, Isl1, Hoxc6, NRSF/REST,* and *Nkx6.1*, were expressed at low or undetectable levels in stage 1 (see Figure 1B–1C), consistent with the reported multipotency of proliferating NSs grown in high glucose [14].

After 2 wk at stage 2, NS-derived cells remained in clusters (see Figure 1A), but Nestin and Ki67 were expressed in fewer than 35% of cells (see Figure 2), indicating possible differentiation of cells toward lineage-restricted fates. Consistent with this view, we detected increased expression of *Nkx6.1, NRSF/REST,* and β-tubulin III (see Figures 1B and 2). We also detected increased expression of *Neurogenin3 (ngn3)* (see Figure 1C), a transcription factor expressed in differentiating neurons, and required for development of pancreatic islet progenitor cells [24,25,26]. *Nkx6.1* and *ngn3* expression were never observed in NS cultures maintained in 37 mM glucose (see Figure 1B; data not shown). These data suggest that glucose restriction initiates developmental programs known to promote differentiation of mammalian neural and neuroendocrine cells. As shown below, insulin expression in NS-derived cells required culture in low-glucose medium at stage 2, but we did not consistently detect insulin^+^ cells by immunohistochemistry until stage 4.

### RA Promotes IPC Development

**Figure 3:**
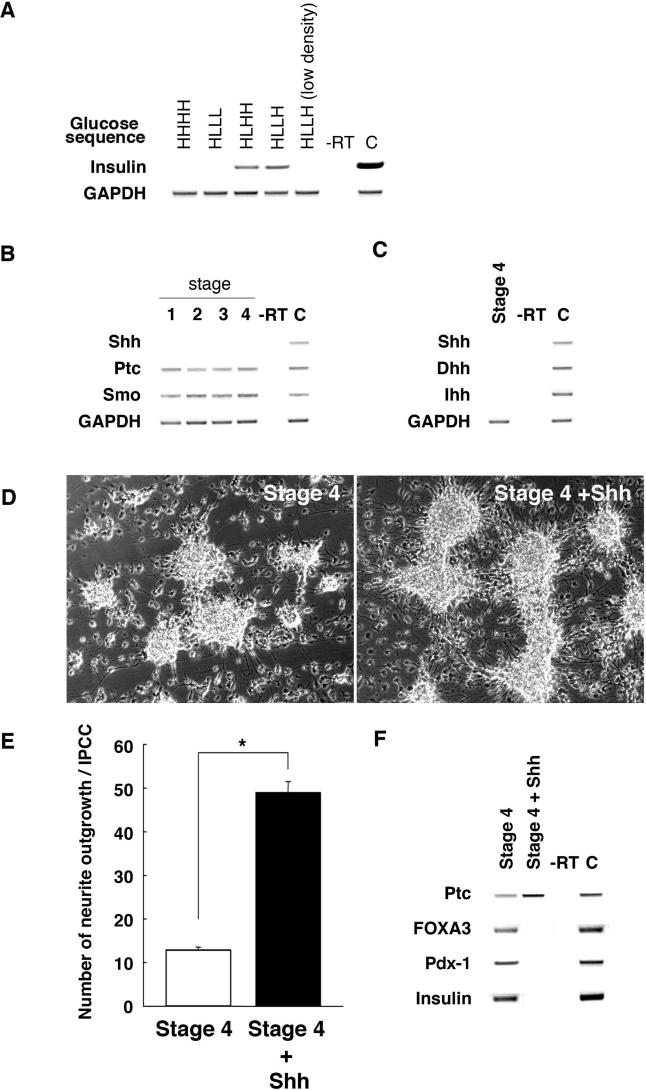
The Sequence of Glucose Concentration Changes, Cell Density, and Absence of Hh Signals Are Essential for Development of IPCs (A) RT-PCR analysis of insulin gene expression for different glucose concentrations and cell densities during stages 2–4. High glucose is designated “H”; low glucose designated “L.” Thus, maintenance of glucose at high concentration in stages 1–4 is abbreviated as “HHHH”; exposure to high glucose in stage 1, followed by reduction of glucose in stages 2–4 is abbreviated “HLLL”; and so forth. Cell clusters analyzed here were cultured at 40–80 clusters/cm^2^ except where indicated (“low density”). Fetal pancreas RNA served as a control (“C”). (B) RT-PCR analysis of Hh signaling factors during stages 1–4 and in fetal spinal cord as a control (“C”). (C) RT-PCR analysis of stage 4 IPC expression of *Shh*, *Desert hedgehog (Dhh)*, and *Indian hedgehog (Ihh)*. Control (“C”) is a spinal cord sample. (D) Neurite outgrowth in control stage 4 IPC clusters, and in stage 4 IPC clusters exposed to 300 nM Shh. Original magnification, 100×. (E) Quantification of neurite outgrowth per control or Shh-treated stage 4 IPC cluster. *, *P* < 0.001. (F) RT-PCR analysis of IPC cluster expression of *Ptc, FoxA3, Pdx1,* and *insulin* following treatment with Shh at stage 4. Control (“C”) is fetal pancreas sample.

RA induces development of primitive endodermal cells from a subset of embryonal carcinoma cell lines, and is an endogenous signal that directs development of posterior organs like the pancreas from embryonic endoderm [27,28]. In vivo exposure to RA is sufficient to induce ectopic development of insulin-expressing tissue in the anterior foregut [27]. Moreover, stage 2 NS-derived cells expressed RA receptors including RAR-α and RAR-γ (see Figure 1C), indicating competence for RA signals. To investigate whether RA could stimulate development of NS-derived cells toward an IPC fate, we measured expression of *Pdx1, Cdx1,* and *FoxA3,* transcription factors that regulate gastrointestinal organ development [29,30]. *Pdx1, Cdx1,* and *FoxA3* expression was induced in “stage 3,” when cell clusters were allowed to adhere to pre-coated wells at a density of 40–80 NSs/cm^2^ and cultured in low-glucose medium with 2 μM RA for 2 wk (see Figure 1A and 1C). We then tested other concentrations of RA and other plating densities. NS cells exposed to 100 nM RA at 20, 40, or 80 NSs/cm^2^ and NS cells exposed to 2 μM RA at a plating density of 20 NSs/cm^2^ did not express these markers or insulin at a later stage (“stage 4”; Figure 3A; data not shown). Thus, higher doses of RA induced markers of posterior fate in NS-derived cells, a dosage response like that seen during RA patterning of posterior gut development in vivo. Moreover, this response was sensitive to plating density, revealing that additional cell-non-autonomous factors may regulate this response. Mesoderm develops in close association with gastrointestinal endoderm both in vivo and in vitro [31,32]. Thus, detection of gut markers like *Pdx1* and *FoxA3* in NS-derived cell cultures raised the possibility that mesodermal cells might co-develop. However, we did not detect expression of known markers of mesoderm formation, including *brachyury, flk-1, myosin light chain-2,* and *β-globin,* at any stage in our cultures (see Figure 1B). By contrast, mesoderm development invariably accompanies endoderm development in embryoid bodies derived from human ES cells or embryonic germ cells [31,32,33]. In stage 3 cultures, Nestin and Ki67 expression was nearly extinguished (see Figure 2), while expression of *En1, ngn3,* and *Hb9* increased (see Figure 1B and 1C). While their numbers increased slightly during stage 3, β-tubulin III^+^ cells composed fewer than 10% of cells in NS-derived clusters (see Figure 2), suggesting that the majority of differentiated cells had acquired non-neural fates. Consistent with this interpretation, we also did not detect expression of homeodomain proteins Hoxc6 or Hoxb9 (see Figure 1B; data not shown), which are induced by RA in spinal cord neuronal precursor cells [21].

RA can activate signaling through the Hedgehog (Hh) pathway, and recent studies show that Hh signals control development of embryonic pancreatic islets in vivo [16,34]. To elucidate the mechanisms underlying RA-induced expression of markers like *FoxA3* and *Pdx1,* we examined Hh signaling during IPC development. We detected expression of the Hedgehog receptors *Patched (Ptc)* and *Smoothened (Smo)* at all stages (Figure 3B), consistent with the possibility that NS-derived cells are competent for Hh signaling. However, we did not detect NS expression of genes encoding Hh ligands like *Shh* (Figure 3B), similar to previous studies [19]. Increased *Ptc* transcription is a known consequence of Shh signaling, but we did not detect changes of *Ptc* expression during stages 1–4, consistent with the absence of detectable expression of Hh ligands. To test directly whether the absence of Hh signaling was required for expression of endodermal or islet markers, including *FoxA3, Pdx1,* and insulin, we added Shh protein to stage 4 cultures, which do not express *Shh* or genes encoding other Hh ligands like Desert hedgehog or Indian hedgehog (Figure 3C). At 300 nM, a dose used to induce neuronal development in ES cell cultures [21], Shh produced a significant increase in neurite outgrowth in NS-derived cells (Figure 3D and 3E; see Methods), and resulted in increased *Ptc* expression (Figure 3F). Moreover, Shh treatment during stage 4 eliminated expression of *Pdx1, FoxA3,* and insulin (Figure 3F). These data are reminiscent of prior studies demonstrating that excess endodermal Shh signaling disrupts pancreas development in vivo [16,34]. Thus, in contrast to the response of ES cells [21], RA treatment of NS-derived cells does not stimulate endogenous Hh signaling. The lack of Hh signals in NS-derived cells blunts neural differentiation and permits differentiation toward endocrine-like cell fates.

### Insulin Expression by IPCs

*ngn3, Pdx1,* and *Hb9* are essential factors for mammalian β-cell development [22], and their expression in stage 3 suggested the potential for deriving IPCs. Insulin expression has not, to our knowledge, been reported in NS-derived cells [19,35]. Moderate hyperglycemia is a potent stimulus for β-cell differentiation and expansion in vivo and in utero [20], so we tested whether exposure of stage 3 cells to elevated glucose levels could induce insulin expression. Semi-quantitative RT-PCR measures of insulin mRNA expression showed that exposure of cultures grown in 5 mM glucose at stage 3 to 17 mM glucose at stage 4 induced insulin expression (Figure 3A). Growth of cells at stage 2 in 5 mM glucose followed by exposure in stages 3 and 4 to 17 mM glucose also resulted in insulin expression at stage 4, but at lower levels. By contrast, no insulin was expressed by cultures maintained in 5 mM glucose or 17 mM glucose throughout stages 2–4 (Figure 3A). Thus, reduction of glucose in stage 2 growth medium followed by a later increase in glucose at stage 3 or 4 was essential for development of IPCs. Insulin-like growth factor-1 may promote insulin secretion and prevent apoptosis of β-cells in vivo [36], and addition of this factor at stage 4 reduced apoptosis approximately 10-fold (see Figure 1A; data not shown). Nicotinamide, which can stimulate in vivo pancreatic endocrine cell differentiation [37], did not affect cell survival, but did increase insulin C-peptide expression approximately 1.5- to 2-fold (data not shown). Thus, simultaneous exposure to high glucose levels, insulin-like growth factor-1, and nicotinamide optimized insulin expression at stage 4. Comparable results were obtained with three independently derived NS lines.

Coinciding with the onset of high insulin expression levels in mouse islets, *ngn3* expression is virtually extinguished [25], while expression of *Pdx1, FoxA3, Isl1,* and *Nkx6.1* in islet β-cells is maintained or increased [22]. Similarly, we noted that IPC maturation from stage 3 to 4 was accompanied by increased insulin expression, reduced *ngn3* expression, and increased or maintained levels of *Isl1, FoxA3,* and *Pdx1* expression (see Figure 1B and 1C). Thus, tissues derived from neural progenitor cells express insulin, *Pdx1,* and *FoxA3,* markers typically co-expressed in foregut-derived tissues. Expression of these markers suggests differentiation of some neural progenitor cells toward an endoderm-like fate. We were unable to detect expression of Pdx1 and FoxA3 using immunohistochemical methods in stage 4 IPCs, (data not shown). We also did not detect expression of Nkx6.1 or islet amyloid polypeptide, markers of mature pancreatic β-cells (see Figure 1C; data not shown). Thus, the sequence of gene expression accompanying formation of IPCs from NSs was similar but not identical to that described for differentiating pancreatic β-cells, and further molecular studies are required to determine the degree of endoderm-like differentiation of cells in IPCs.

**Figure 4:**
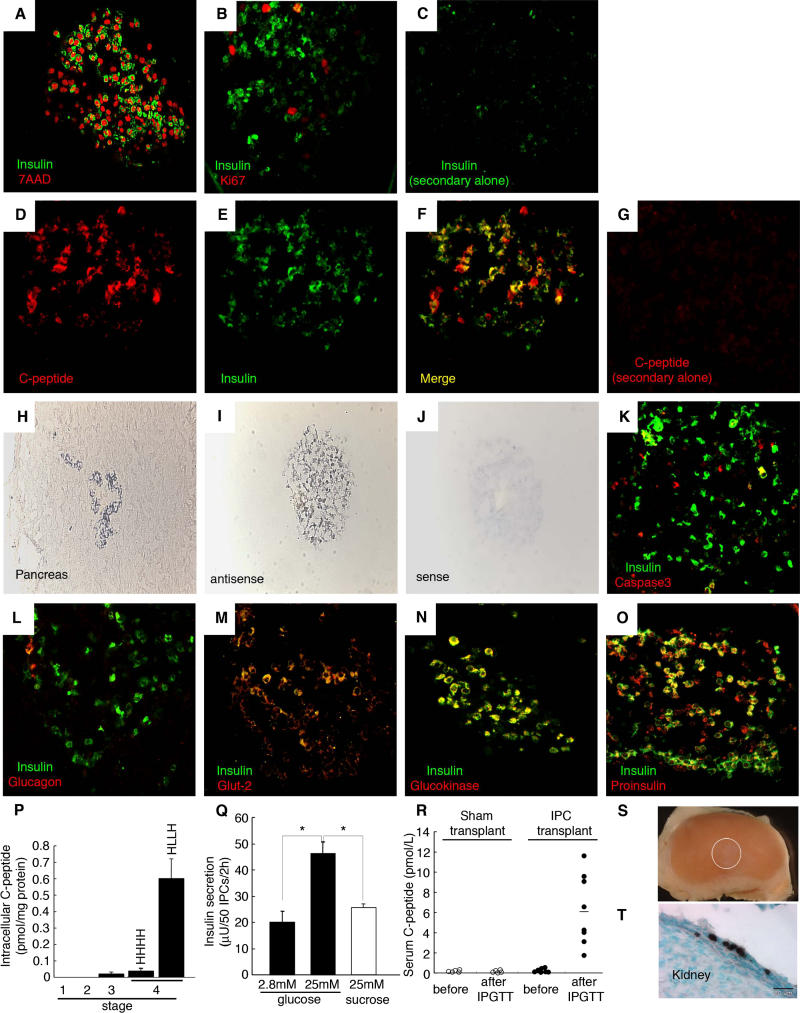
Stage 4 IPCs Express Characteristic Pancreatic β-Cell Markers and Are Glucose-Responsive (A–C) Immunofluorescent images of stage 4 IPCs were obtained by confocal microscopy and are representative of at least ten samples for each probe. Shown is simultaneous immunofluorescent staining of insulin and either the nuclear marker 7AAD (A) or Ki67 (B). (C) Lack of immunostaining upon omission of the anti-insulin primary antibody. (D–G) Detection of insulin C-peptide in cells stained by anti-insulin antibodies. There is complete overlap between immunostained C-peptide and insulin in the cytoplasm. (H and I) Representative in situ hybridization with antisense riboprobes specific for human insulin on sectioned human fetal pancreatic islet cells (H) or a stage 4 IPC cluster (I). (J) Sense control probe in situ hybridization on a stage 4 IPC cluster. (K–O) Simultaneous immunofluorescent detection of insulin and cleaved caspase-3 (K), glucagon (L), Glut-2 (M), glucokinase (N), or proinsulin (O). (P) Intracellular C-peptide content of IPCs during stages 1–4 by human C-peptide-specific ELISA. (Q) In vitro secretion assay of stage 4 IPCs. Insulin release followed a step increase from 2.8 mM to 25 mM glucose, but not following exposure to 25 mM sucrose, which served as an osmotic control. *, *p* < 0.001. (R) Serum human C-peptide detection 2-wk after sham or IPC cluster transplantation in NOD *scid* mice. Samples were obtained before or 30 min after intraperitoneal glucose challenge (IPGTT). (S) Lack of tumor growth after renal transplantation of stage 4 IPCs (graft indicated by white circle). (T) Immunohistochemical detection of human C-peptide expression in stage 4 IPCs (cells stained brown) 2-wk after engraftment; bar is 20 μm. Original magnification of (H–J) and (T) was 250×. All other photomicrographs' original magnification was 630×.

**Figure 5:**
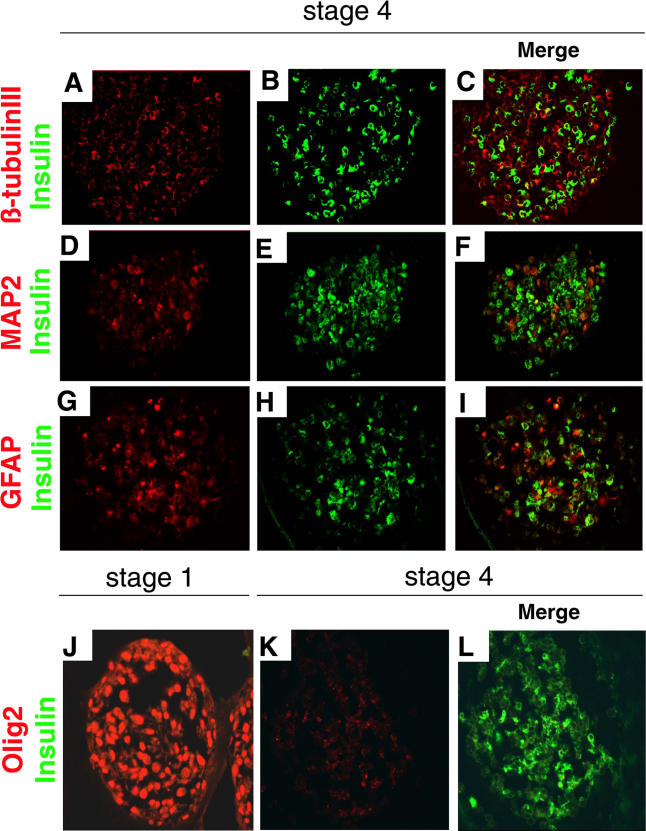
Stage 4 IPCs Do Not Express Markers of Differentiated Neural Cells (A–C) Immunohistochemical detection of β-tubulin III (A) and insulin (B) in stage 4 cells, and a merge of both images (C). (D–F) Immunohistochemical detection of MAP2 (D) and insulin (E) in stage 4 cells, and a merge of both images (F). (G–I) Immunohistochemical detection of GFAP (G) and insulin (H) in stage 4 cells, and a merge of both images (I). (J–L) Immunohistochemical detction of Olig2 at stage 1 (J) and stage 4 (K) and merged view of Olig2^+^ and insulin^+^ cells at stage 4 (L). Original magnification was 630×.

We detected expression of insulin protein by immunohistochemistry in 90% of stage 4 cell clusters (Figure 4). An average of 26% of cells in stage 4 clusters were insulin^+^, and appeared healthy, with abundant cytoplasm and a well-defined nucleus delineated by the nuclear stain 7AAD (Figure 4A). Of the cells composing stage 4 clusters, 45% were β-tubulin III^+^, none of which contained insulin (Figure 5A–5C). Insulin^+^ cells showed little or no co-expression of neural stem cell markers like Nestin (see Figure 1), or other markers of neural lineages known to develop from human neural progenitors, like MAP2 (differentiated neurons; Figure 5D–5F), Olig2 (multipotent precursors, bipotent glial precursors, and oligodendrocytes; Figure 5J–5L; see [38]), or myelin basic protein (data not shown). Occasional rare insulin^+^ cells expressed detectable GFAP (a marker of astrocytes; Figure 5G–5I). Thus, while we cannot completely rule out the possibility that some neural cell types expressed insulin, these data suggest that nearly all insulin^+^ cells produced in stage 4 cultures were non-neuronal.

Nuclei in insulin^+^ cells were not stained by an antibody to Ki67 (see Figures 2 and 4B), suggesting that insulin^+^ cells at stage 4 are post-mitotic, like mature pancreatic β-cells. Immunostaining with specific antibodies revealed proinsulin and C-peptide, an internal portion of the proinsulin translation product, in the cytoplasm of all insulin^+^ cells (see Figure 4D–4G and 4O), supporting our conclusion that insulin protein was produced in stage 4 IPCs. In situ hybridization with antisense riboprobes specific for human insulin mRNA labeled 25%–30% of stage 4 cells (see Figure 4H–4J), providing further evidence of insulin production. We observed only rare insulin^+^ cells stained by antibodies specific for activated caspase-3 (<0.3%; see Figure 4K) or TUNEL assay (data not shown). In stage 4 cell clusters we did not consistently detect expression of glucagon (see Figure 4L), pancreatic polypeptide, or somatostatin by immunohistochemistry. The absence of glucagon gene expression in our IPC cultures was further confirmed by RT-PCR (data not shown). Thus, like in pancreatic islets, insulin was the principal hormone produced by IPCs. However, the composition of IPCs was distinct from that of pancreatic islets, and further studies are required to determine the basis for this difference.

To quantify insulin expression in IPCs, we used a human insulin C-peptide-specific ELISA (see Figure 4P). By ELISA, we did not detect C-peptide in stage 3 or stage 4 culture media, which contained supplementary bovine insulin. Thus, use of a C-peptide assay did not detect medium-derived bovine insulin in measures of IPC insulin content [39]. C-peptide levels at stages 1–3 were low or undetectable (see Figure 4P), consistent with the lack of immunostainable insulin (see Figure 2) in cells at these earlier stages. Similarly, in cultures maintained in 17 mM glucose during stages 1–4 (“HHHH”), C-peptide levels were not detectable (see Figure 4P). In cell clusters exposed to 5 mM glucose in stages 2–3 then switched to 17 mM glucose at stage 4 (“HLLH”), insulin C-peptide levels were 0.36 fmol per cluster, and the average number of C-peptide^+^ cells per cluster was 200. Thus, we estimate 0.0018 fmol C-peptide per NS-derived IPC. A single β-cell contains approximately 0.6 fmol C-peptide and insulin [40], so we calculate that insulin C-peptide content in one stage 4 IPC is approximately 0.3% of the level in isolated human β-cells. Collectively, these experiments confirm that IPCs transcribe and translate insulin, and rule out that insulin measured in IPCs is derived from medium, as shown in another system [39].

### IPCs Are Glucose Responsive

Glucokinase and glucose transporters like Glut-2 are essential regulators of glucose responses in pancreatic β-cells, and expression of these regulators in stage 4 insulin^+^ cells (see Figures 1C, 4M, and 4N) suggested that IPCs could sense and respond to glucose. To test whether IPCs respond appropriately to glucose stimulation, we measured insulin release in static batch in vitro assays. We found that IPC insulin release increased markedly following a step increase of glucose from 2.8 to 25 mM (see Figure 4Q). To examine whether IPCs are similarly responsive to glucose stimulation in vivo, we performed a series of IPC grafting experiments in immunocompromised recipient mice. Two weeks following engraftment (1,000 IPC-containing clusters/mouse), recipient mice and sham-transplanted controls were fasted overnight, and human C-peptide levels measured. Human C-peptide was undetectable in mouse sera prior to glucose challenge, or following glucose challenge of sham-transplanted controls (see Figure 4R). In contrast, 30 min after intraperitoneal glucose challenge of IPC recipients we detected 6.1 ± 1.2 pmol/l human C-peptide (*p* < 0.001), approximately 0.5%–1% of serum C-peptide levels observed following engraftment of 2,000 human islet equivalents in a prior study [41]. Thus, IPCs respond to glucose challenge in vivo by releasing insulin C-peptide. Analysis of IPC graft sites 4 wk after transplantation revealed nests of transplanted cells without obvious tumor formation. Histologic analysis and immunohistochemical detection revealed that C-peptide-expressing cells persisted in the graft site (see Figure 4S and 4T). Thus, engrafted IPCs remained differentiated and survived up to a month following transplantation.

## Discussion

There is widespread interest in developing tissue replacement strategies for treatment of human disorders like diabetes mellitus and Parkinson disease. While much attention has been focused on the promise of ES cells for tissue replacement, recent work suggests that neural stem cells, like ES cells, may have an unusually broad differentiation potential. For example, Gage and colleagues demonstrated the conversion of mouse neural stem cells to the endothelial lineage, indicating that plasticity is a bona fide property of cultured neural stem cells [42]. These results were also unexpected because endothelial cells and neuronal cells normally derive, respectively, from mesoderm and ectoderm, distinct embryonic germ layers. Here we show that human neural stem cells have a similarly broad differentiation potential, and that specific in vitro culture conditions can divert neural stem-derived cells from neural lineages toward a fate with endocrine and endodermal characteristics.

Our study shows that application of endogenous signals governing pancreas development to human neural progenitor cells can generate glucose-responsive IPCs. Systematic variation of the identity, concentration, and sequence of these signals led to discovery of methods culminating in IPC formation. However, there are limitations to our findings that should be noted. First, our molecular analysis of differentiating IPCs showed that they remain distinct from mature pancreatic islet β-cells; in other words, the insulin^+^ cells derived by our methods are not mature β-cells. For example, reverse transcriptase polymerase chain reaction confirmed that IPCs were enriched for a combination of gene products that approximate those expressed in developing pancreatic islet cells. However, the temporal sequence of expression of some of these products, like glucokinase, Glut-2, and Pdx1 does not precisely recapitulate that observed in the embryonic pancreas (reviewed in [22]), and transcription of other genes typically expressed in β-cells, like *Nkx6.1,* was not detected in later stage IPCs (see Figure 1). Thus, the insulin^+^ cells derived in this study are not bona fide β-cells. Further experimentation may elucidate the basis for these differences, revealing the extent of neural progenitor cell development toward an endocrine cell fate. Recent studies suggest that neural cells can be derived from adult pancreatic epithelium [7,8], adding to a growing body of data demonstrating numerous similarities in neural and pancreatic endocrine development (reviewed in [43]). In light of these similarities, we speculate that methods leading to the production of β-cell factors like Nkx6.1 in stage 4 clusters may enhance the β-cell-like qualities of neural-progenitor-derived insulin^+^ cells, and thereby permit differentiation of tissues that more closely resemble endoderm-derived islet cells. Second, our immunohistochemical analysis shows that stage 4 insulin-expressing cells do not express markers like Nestin, β-tubulin III, MAP2, or Olig2, suggesting that these insulin^+^ cells are non-neuronal, but we cannot formally rule out the alternate possibility that our methods produced neural cells capable of secreting insulin. Third, we have not demonstrated fully that the stimulus–secretion coupling apparatus in IPCs is similar to that in pancreatic islets. While insulin release by IPCs produced here appears to be glucose-sensitive, both in vitro and in vivo, future work should elaborate whether IPC insulin is stored in dense-core secretory vesicles, and whether secretogogues or secretion potentiators other than glucose (like amino acids or sulfonylureas) stimulate insulin release by IPCs, like in pancreatic islets. Lastly, we have not ameliorated glucose regulation in diabetic animal models with the human IPCs described here. Serum levels of insulin C-peptide expression achieved following IPC transplantation in glucose-challenged mice were less than 1% of normal (see Figure 4). Thus, we did not attempt IPC transplantations in overtly diabetic animals, since prior studies suggest that insulin production at 10% of normal (or greater) may be required to improve glucose regulation in diabetic patients and animal models [10,41]. Additional studies to test the impact of transplanted IPCs in animal models of diabetes therefore await production of IPCs capable of insulin secretion at levels higher than achieved here.

Nevertheless, compared to other methods for IPC development from human stem cells [13,44], our methods produced insulin at the highest levels yet achieved from an expandable, human stem-cell-derived tissue. Multiple experimental approaches were taken to demonstrate that these IPCs transcribe, translate, and secrete insulin, and to rule out the possibility that insulin measured in IPCs derived from the culture media. In vitro studies demonstrate that IPCs release insulin in a glucose-responsive manner, like islets. Moreover, IPCs transplanted into mice remained differentiated and released circulating human insulin in a glucose-dependent manner. Thus, for the first time, we demonstrate moderately efficient production of glucose-responsive IPCs from an expandable population of human stem cells. Current islet transplantation methods require an estimated 5 × 10^8^ to 10^9^ β-cells per recipient [45]. From 10^6^ cells at stage 2, we produced an average of 200–400 clusters with approximately 2,000 cells per cluster; approximately 25% of these cells are C-peptide^+^ and release 0.5%–1% of C-peptide secreted by β-cells. Based strictly on these yields, we would need to expand IPC production approximately 10^5^-fold to meet the need for one transplantation. Undifferentiated NSs can be readily expanded through at least a dozen passages [14], suggesting sufficient cell numbers could be generated to “scale up” this protocol for transplant-based therapies.

We did not exhaust all possible factor combinations in our study: we speculate that further method refinements may improve the efficiency of NS conversion into IPCs, as well as IPC insulin synthesis and stimulus–secretion coupling, the hallmark functions of mature β-cells. If so, then human neural stem cells may serve as a valuable model for elucidating the mechanisms and factors that regulate neuroendocrine cell differentiation. For instance, addition of glucagon-like peptide-1, TGF-β ligands, or other factors that potentiate β-cell maturation, growth, and insulin secretion [46,47,48] may improve the methods described here. Because our method is based solely on extracellular factor modulation, in the absence of genetic manipulations, it could serve as the basis for developing replacement islets from a wide range of human stem cells, including neural stem cells and ES cells.

## Supporting Information

### Accession Numbers

The National Center for Biotechnology Information (www.ncbi.nlm.nih.gov/) accession numbers for the genes and gene products discussed in this paper are *brachyury* (AF012130), *Cdx1* (NM_000209), Desert hedgehog (NM_021044), *En1* (NM_001426), *flk-1* (AF035121), *FoxA3* (NM_004497), GAPDH (NM_002046), GFAP (NM_002055), glucokinase (M90299), *Hb9* (NM_005515), *Hoxb9* (NM_024017), *Hoxc6* (NM_004503), human insulin cDNA (J00265), Indian hedgehog (NM_002181), *Isl1* (NM_002202), MAP2 (U01828), myelin basic protein (M13577), *myosin light chain-2* (X57542), Nestin (NM_006617), *ngn3* (AF234829), *Nkx6.1* (NM_006168), *NRSF/REST* (U13879), Olig2 (NM_005806), *Pdx1* (NM_000209), *Ptc* (U59464), RAR-α (NM_000964), RAR-β (BC060794), RAR-γ (NM_000966), *Shh* (L38518), *Smo* (AH007453), *β-globin* (NM_000518), and β-tubulin III (BC000748).

**Patient Summary**

**Background**

The transplantation of insulin-secreting cells has proved effective in treating severe cases of type 1 diabetes, but currently such cells have to be collected from donors after death. The process of isolation is very laborious and does not provide sufficient numbers of cells to permit wider use of this transplant-based treatment. A renewable source of these cells would be very valuable. Previous work has shown that some nerve cells can also produce insulin.

**What Did the Researchers Do?**

They took stem cells that had come from human brains and treated them with a variety of different substances, including glucose, to see if they could alter the way the cells normally develop. They managed to manipulate the stem cells so that they developed into more mature cells that secreted insulin, and could respond to glucose. They put the cells into mice, and found that there, too, they could respond to glucose by producing insulin, albeit at very low levels.

**What Do the Results Mean for Patients?**

These findings are a first step towards producing a renewable source of insulin-producing cells; however, the amount of insulin produced was quite low. In addition much more work will need to be done to ensure the safety of the procedure over the long term.

**Where Can I Get More Information?**

The National Diabetes Information Clearinghouse, part of the National Institute of Diabetes and Digestive and Kidney Diseases has an information page on islet cell transplantation: http://diabetes.niddk.nih.gov/dm/pubs/pancreaticislet/

Diabetes UK, a charity, also has information: http://www.diabetes.org.uk/islets/

## Abbreviations

ELISA: enzyme-linked immunosorption assay
ES: embryonic stem
Hh: Hedgehog
IPC: insulin-producing cell
NS: neurosphere
RA: retinoic acid
Shh: Sonic hedgehog
